# Recombinant protein production provoked accumulation of ATP, fructose-1,6-bisphosphate and pyruvate in *E. coli* K12 strain TG1

**DOI:** 10.1186/s12934-021-01661-9

**Published:** 2021-08-26

**Authors:** Jan Weber, Zhaopeng Li, Ursula Rinas

**Affiliations:** 1 Helmholtz Centre for Infection Research, Inhoffenstraße 7, 38124 Braunschweig, Germany; 2 Technical Chemistry-Life Science, Leibniz University of Hannover, Hannover, Germany

**Keywords:** *Escherichia coli*, Metabolic burden, Recombinant protein production

## Abstract

**Background:**

Recently it was shown that production of recombinant proteins in *E. coli* BL21(DE3) using pET based expression vectors leads to metabolic stress comparable to a carbon overfeeding response. Opposite to original expectations generation of energy as well as catabolic provision of precursor metabolites were excluded as limiting factors for growth and protein production. On the contrary, accumulation of ATP and precursor metabolites revealed their ample formation but insufficient withdrawal as a result of protein production mediated constraints in anabolic pathways. Thus, not limitation but excess of energy and precursor metabolites were identified as being connected to the protein production associated metabolic burden.

**Results:**

Here we show that the protein production associated accumulation of energy and catabolic precursor metabolites is not unique to *E. coli* BL21(DE3) but also occurs in *E. coli* K12. Most notably, it was demonstrated that the IPTG-induced production of hFGF-2 using a *tac*-promoter based expression vector in the *E. coli* K12 strain TG1 was leading to persistent accumulation of key regulatory molecules such as ATP, fructose-1,6-bisphosphate and pyruvate.

**Conclusions:**

Excessive energy generation, respectively, accumulation of ATP during recombinant protein production is not unique to the BL21(DE3)/T7 promoter based expression system but also observed in the *E. coli* K12 strain TG1 using another promoter/vector combination. These findings confirm that energy is not a limiting factor for recombinant protein production. Moreover, the data also show that an accelerated glycolytic pathway flux aggravates the protein production associated “metabolic burden”. Under conditions of compromised anabolic capacities cells are not able to reorganize their metabolic enzyme repertoire as required for reduced carbon processing.

## Background

Recombinant protein production in *Escherichia coli* is frequently associated with an impact on the host cell metabolism known as “metabolic burden”. The extent of the metabolic burden depends on the properties of the recombinant gene, in particular on the properties of the encoded transcript and protein product [1], the environmental conditions during production but also on the host/expression vector combination. Frequently, the protein production associated metabolic burden becomes evident through growth inhibition and enhanced acetate formation [2, 3].

Until lately it was common understanding that the recombinant protein production associated metabolic burden is the result of redirection of metabolites and energy away from host cell towards plasmid encoded functions. More recently, it was shown for the *E. coli* BL21(DE3)/T7 promoter expression vector combination that the growth inhibitory effect of recombinant protein production is not elevated energy and precursor withdrawal for plasmid encoded functions but, on the opposite, ample catabolic generation and insufficient withdrawal of energy and precursor for anabolic purposes [4]. The growth inhibitory effect of recombinant protein production has been shown to be mainly attributable to recombinant gene transcription but also partly to recombinant protein synthesis in particular when difficult-to-fold proteins are being produced [5, 6]. There is also increasing evidence that the growth inhibitory effect of accumulating recombinant RNA depends on the sequence with some RNA sequences being more toxic than others [6]. So far, the exact molecular mechanism of their interference with cellular growth and anabolic functions is not yet known.

For the *E. coli* BL21(DE3)/T7 promoter expression vector combination it has been shown that induced recombinant gene expression leads to ATP accumulation which is being further subjected to degradation pathways finally leading to the extracellular accumulation of hypoxanthine [4]. This has been shown for a variety of recombinant genes and proteins including also for the production of human basic fibroblast growth factor (hFGF-2). Thus, production of hFGF-2 using the BL21(DE3)/T7 promoter expression vector combination is connected to energy excess and clearly not to energy limitation. On the other hand, contradicting results concerning the energetic status of producing cells have been obtained during temperature-induced production of hFGF-2 using the *E. coli* K12 strain TG1 where it was shown that the ATP level as well as the adenylate energy charge decreased in response to temperature-induced (shift to 42 °C) production [7, 8].

To resolve these seemingly contradicting findings concerning the energetic status of producing cells, the production of human basic fibroblast growth factor was carried out in the *E. coli* K12 strain TG1 using a temperature-independent, IPTG-inducible *tac*-promoter based expression system. These experiments should provide additional information if excessive energy formation and insufficient energy withdrawal for anabolic purposes could be a more general phenomenon during recombinant protein production that can be generalized to other *E. coli* strains and expression systems. Thus, we analyze here the metabolic response towards production of hFGF-2 in the *E. coli* K12 strain TG1 using an IPTG-inducible *tac*-promoter based expression system with special emphasis on the growth inhibitory effect of hFGF-2 production and the energetic status of the cells. Moreover, we pay special attention to the time course data of carbon overflow metabolites such as acetate and pyruvate as well as to time-course data of glycolytic pathway intermediates after induction of hFGF-2 synthesis. In all studies on the metabolic stress response towards induced production of hFGF-2 utilizing different host strain/expression vector combinations the same codon-optimized gene encoding hFGF-2 was used.

## Results

Production of hFGF-2 synthesis was carried out in the *E. coli* K12 strain TG1 using a *tac*-promoter controlled expression system in batch cultures on defined medium with glucose as carbon substrate. A control cultivation was performed using the *E. coli* K12 strain TG1 transformed with the parental plasmid not containing a gene insert downstream of the *tac*-promoter.

### IPTG-induced production of hFGF-2 in *E. coli* TG1:pJHLbFGF leads to accumulation of pyruvate and inhibition of growth and respiratory activity

**Fig. 1:**
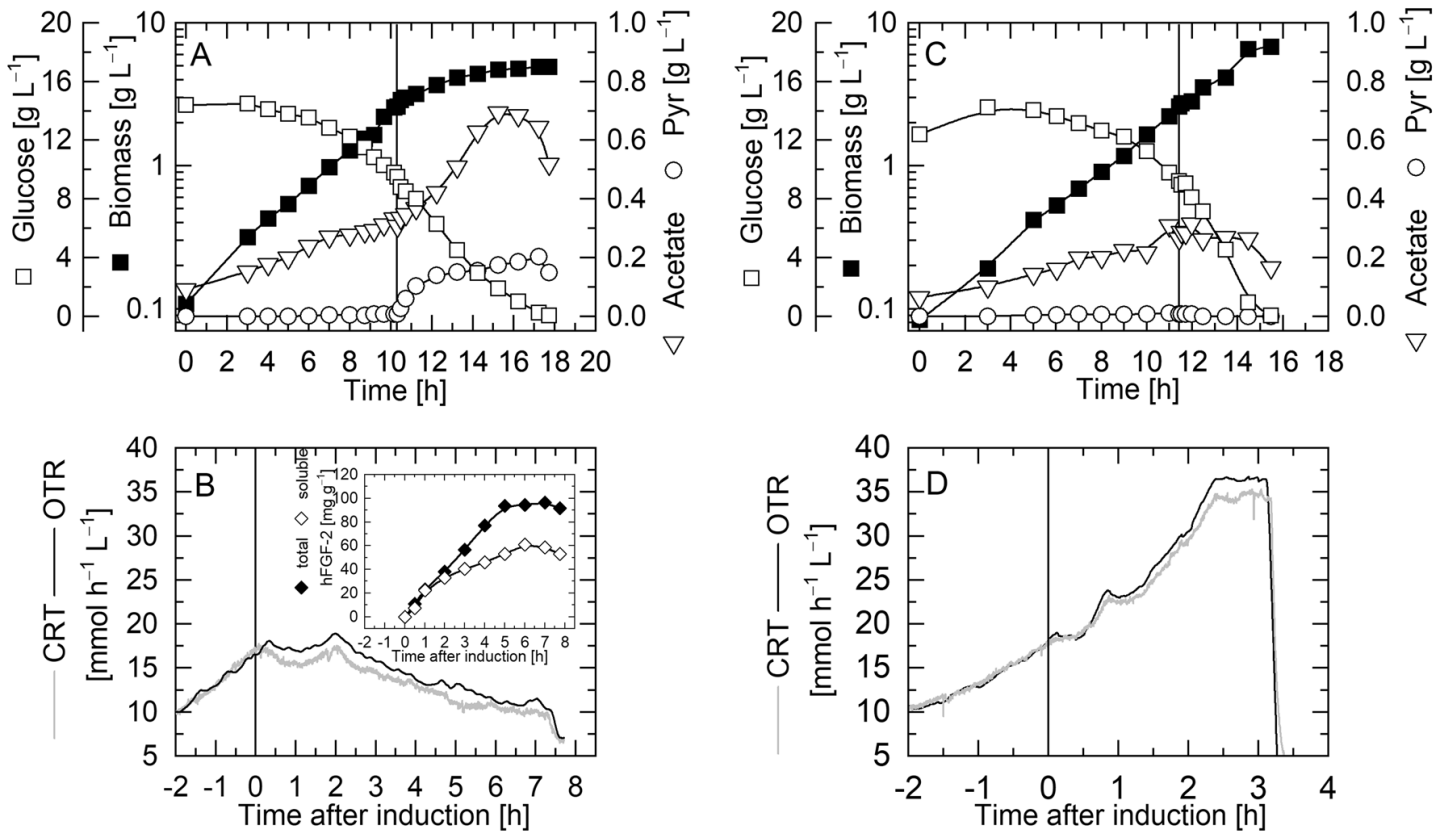
Growth inhibition in response to IPTG-induced production of hFGF-2 in batch culture. Time-course data of biomass, glucose, acetate and pyruvate concentrations and respiratory activity, volumetric carbon dioxide and oxygen transfer rates (**A**, **B**) during production of hFGF-2 with *E. coli* TG1:pJHLbFGF and (**C**, **D**) in the non-producing control culture with *E. coli* TG1:pJHLΔ growing under identical conditions. The insert in **B** depicts the time course of target protein production (total and soluble concentrations of hFGF-2 per biomass). The vertical lines indicate the time point of IPTG addition

Induction of hFGF-2 synthesis was immediately followed by accumulation of pyruvate in the culture medium (Fig. 1A). Inhibition of growth and respiratory activity, however, occurred with a short delay of approx. 30 min after IPTG addition (Fig. 1A, B). In the control culture of *E. coli* TG1:pJHLΔ neither inhibition of growth and respiratory activity nor accumulation of pyruvate were detectable after IPTG addition (Fig. 1C, D). Moreover, glucose consumption was slower after IPTG addition in the culture producing hFGF-2 compared to the induced control culture not carrying a recombinant gene downstream of the *tac*-promoter (Fig. 1A, C). Acetate formation was observed in both cultures, however, a stronger increase occurred upon IPTG addition in the culture producing hFGF-2 (Fig. 1A, C).

### Increase of ATP and adenylate energy charge in response to IPTG-induced production of hFGF-2

**Fig. 2:**
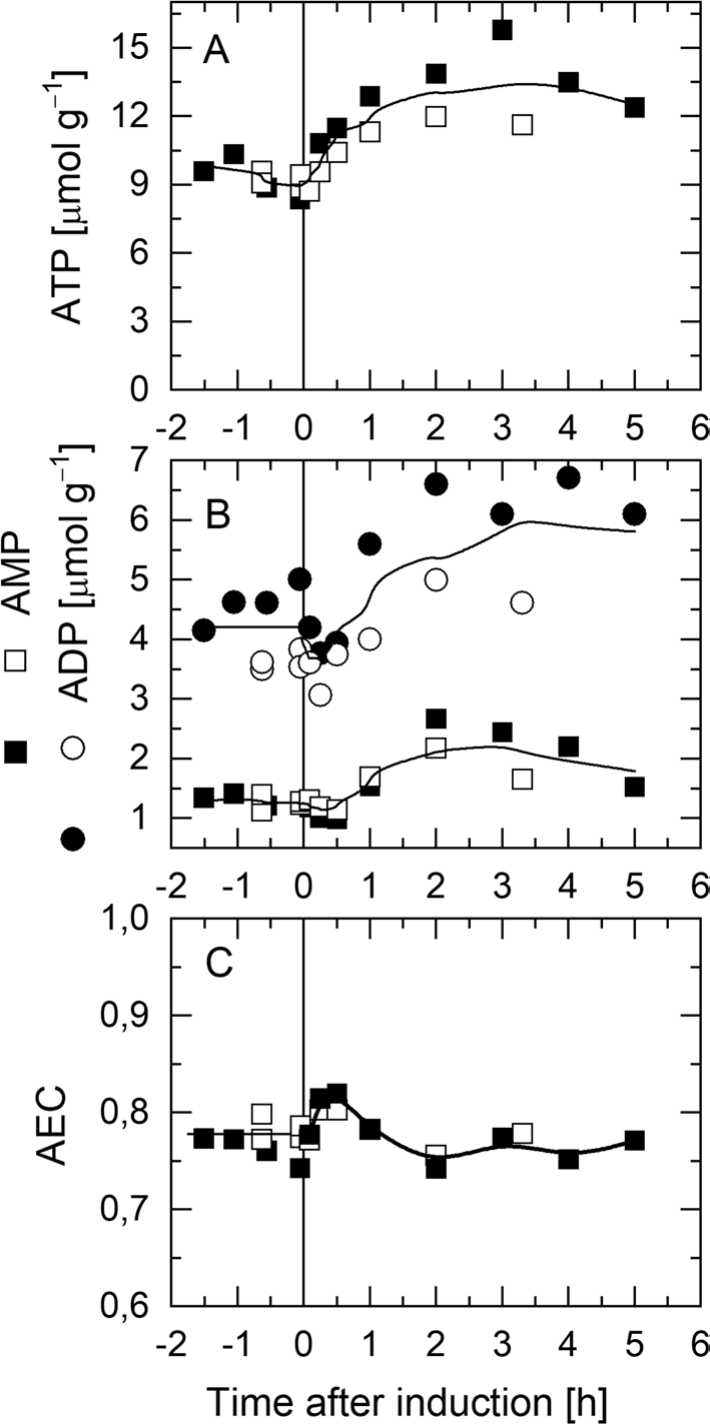
Energetic status of *E. coli* TG1:pJHLbFGF during IPTG-induced production of hFGF-2. Time-course data of specific concentrations of **A** ATP, **B** ADP and AMP and **C** the adenylate energy charge (AEC) during production of hFGF-2. Open and closed symbols correspond to two independent cultivations (trend line serves for better visualization). The vertical lines indicate the time point of IPTG addition

After induction of hFGF-2 synthesis ATP levels increased instantaneously and remained high for the entire production phase (Fig. 2A). The ATP increase was followed by an approx. one hour delayed but also persistent increase in the concentrations of the ATP degradation products ADP and AMP (Fig. 2B). The adenylate energy charge revealed an instantaneous but transient increase and reached approx. one hour after IPTG addition again the pre-induction level (Fig. 2C). These data show that ATP is clearly not limiting host cell proliferation after induced synthesis of hFGF-2. On the contrary excess of ATP is subjected to degradation pathways leading to accumulation of ADP and AMP. The data also show that cells try to maintain energetic homeostasis aiming for a constant adenylate energy charge.

### The key regulatory molecule fructose-1,6-bisphoshate strongly increases in response to IPTG-induced production of hFGF-2

**Fig. 3:**
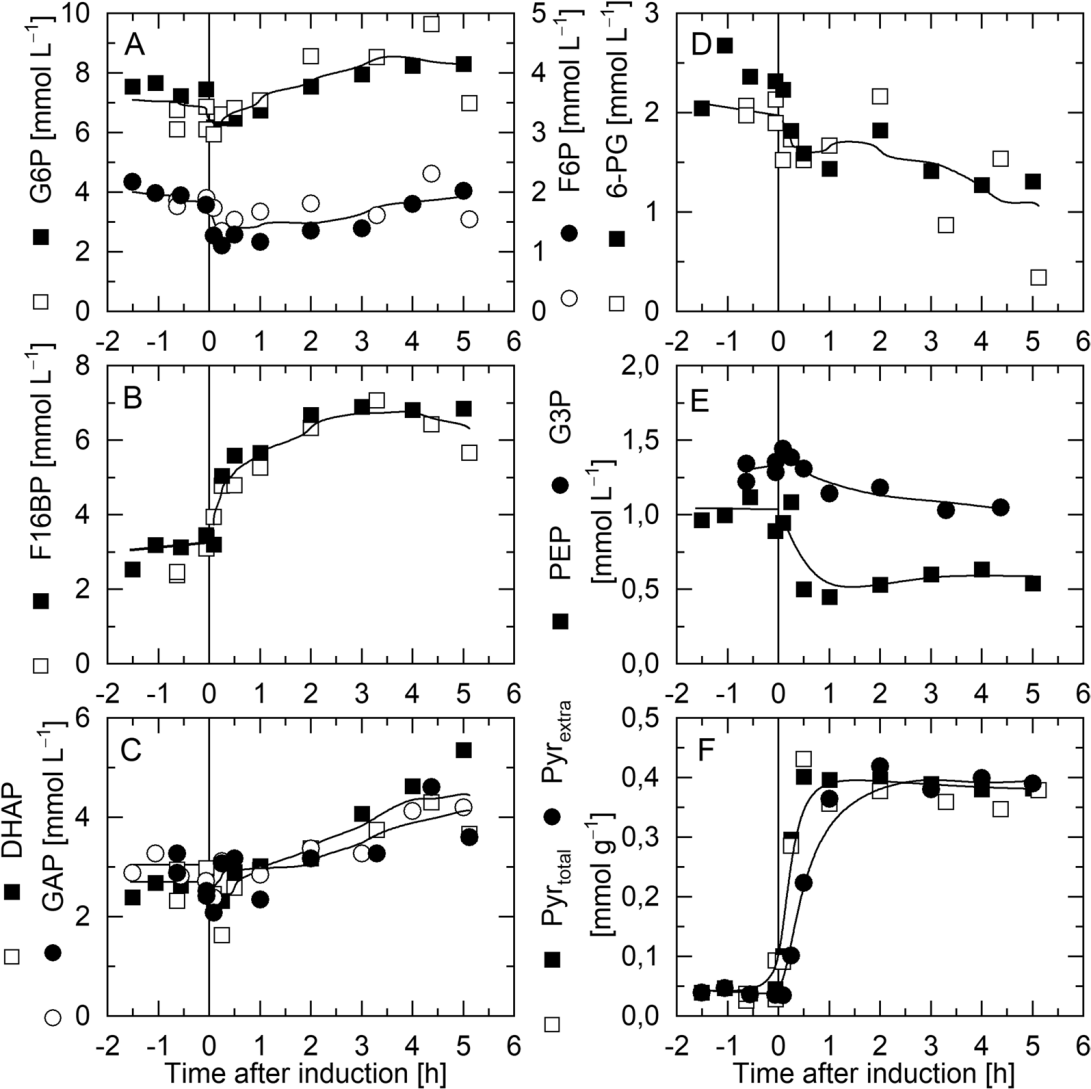
Glycolytic pathway intermediates in *E. coli* TG1:pJHLbFGF during IPTG-induced production of hFGF-2. Time-course data of **A** glucose-6-phosphate (G6P) and fructose-6-phosphate (F6P), **B** fructose-1,6-bisphosphate (F16BP), **C** dihydroxyacetonphosphate (DHAP) and glyceraldehyde-3-phosphate (GAP), **D** 6-phosphogluconate (6PG, pentose phosphate pathway intermediate), **E** 3-phosphoglycerate (3PG) and phosphoenolpyruvate (PEP) and **F** intra- and extracellular pyruvate concentrations. Open and closed symbols correspond to two independent cultivations (trend line serves for better visualization). Please note that the volumetric concentrations given relate to the concentrations in the *E. coli* cytoplasm. The vertical lines indicate the time point of IPTG addition

The time dependent changes of the intracellular concentrations of upper and lower glycolytic pathway metabolites as well as pyruvate and the pentose-phosphate pathway metabolite 6-phosphogluconate (6PG) were followed before and after IPTG-induced production of hFGF-2 (Fig. 3). Prior to induction, metabolite pools remained constant. Major changes occurred within the first 30 min after IPTG addition followed by slower adjustments and stabilization at the new metabolite pool levels. The most striking changes after initiation of hFGF-2 production involved a strong and persistent increase in the intracellular concentration of fructose-1,6-bisphosphate (> 100% increase of F16BP, Fig. 3B) and a strong but transient increase of the intracellular pyruvate concentrations followed by pyruvate excretion into the culture medium (> 700% increase of extracellular pyruvate, Fig. 3F). The changes in the concentrations of the other metabolites were not that prominent except for the notable decrease in the concentration of intracellular phosphoenolpyruvate (PEP) (approx. 50% decrease, Fig. 3E).

## Discussion

Recently it was shown that the growth inhibitory effect of IPTG-induced human hFGF-2 production in *E. coli* BL21(DE3) using a pET-based expression vector reflects similarities to a carbon overfeeding response and is the result of constraints in anabolic pathways and accompanied by accumulation of ATP and ATP degradation products as well as primary metabolites such as pyruvate [4]. Those studies are complemented here by analyzing the cellular response towards IPTG-induced production of hFGF-2 in the *E. coli* K12 strain TG1.

### Energy excess during recombinant protein production also in *E. coli* K12 strain

As observed for IPTG induced hFGF-2 production using *E. coli* BL21(DE3) and the pET-based expression system, we also detect growth inhibition, reduced respiratory activity, enhanced acetate formation and accumulation of pyruvate and ATP in response to IPTG-induced production of hFGF-2 employing the *E. coli* K12 strain TG1 in combination with *tac*-promoter controlled recombinant gene expression. These findings clearly show that energy excess during recombinant protein production is not unique for the BL21(DE3)/T7 promoter expression vector combination but also observed in *E. coli* K12 strains consolidating the hypothesis that the “metabolic burden” of recombinant protein production is not energy limitation caused by elevated energy demand for plasmid encoded functions. Thus, the recombinant gene expression associated “metabolic burden” can be related to restrictions in anabolic functions and not to restrictions in catabolic energy generation. The seemingly contradicting findings of a decrease in the intracellular ATP content as well as in the adenylate energy charge in response to temperature-induced production of hFGF-2 [7, 8] combined with increased respiratory activity [8, 9] originally interpreted as elevated energy withdrawal for plasmid encoded functions is presumably just the result of a generally higher energy demand and/or spilling at non-physiologically elevated temperatures.

### Energy generation is not a limiting factor for protein production and cellular growth

An immediate but transient increase in the intracellular ATP content in the time scale of seconds was also observed for non-recombinant *E. coli* in response to a glucose pulse [10]. Moreover, an instantaneous and transient ATP increase was found in non-recombinant *E. coli* TG1, the strain employed in this study for the production of hFGF-2, after a carbon-upshift (dilution rate upshift in glucose-limited continuous cultures, 30 °C) together with a transient burst in respiratory activity [11]. This carbon upshift experiment also revealed that the sudden burst in respiratory activity and ATP content was immediately followed by a decrease in the ATP level and the adenylate energy charge, which both returned within minutes to a new steady state level concomitant to a decrease in the respiratory activity [11]. These data together with the analyses presented here suggest that respiratory ATP generation is not the bottleneck neither limiting regular growth not the execution of plasmid encoded functions. These data even indicate that *E. coli* is adapted to instantaneously increase respiratory ATP generation under conditions of “sudden and unexpected” carbon supply but can also quickly reduce respiratory energy generation if energy utilization cannot keep pace with ATP formation. As these adaptations occur within seconds and minutes they are certainly controlled through allosteric regulation of metabolic enzyme activity most likely through (an) energetic status sensing molecule(s).

### FBP accumulation during protein production indicates elevated glycolytic pathway flux

In addition to the analysis of the respiratory activity and energetic cell status, the time-course data of glycolytic pathway intermediates were determined after IPTG induced production of hFGF-2. These analyses revealed a persistent increase in the level of fructose-1,6-bisphosphate (FBP) and pyruvate as well as a decrease in the phosphoenolpyruvate (PEP) level in response to hFGF-2 production (Fig. 3). These findings are consistent with a permanently elevated flux through the glycolytic pathway during the production period. FBP is known as a glycolytic flux sensor [12], more precisely, FBP senses the flux through the upper glycolytic pathway [13]. FBP can modify the actvity of downstream enzymes of the glycolytic pathway, namely pyruvate kinase and PEP carboxylase through allosteric regulation [14]. FBP accumulates until enzyme activity in the lower part of glycolysis matches with the upper glycolytic pathway flux [15]. Thus, a high FBP level in combination with a low PEP to pyruvate ratio, as observed here during IPTG induced hFGF-2 production, indicates elevated glycolytic flux accelerated through allosteric feed-forward activation through FBP on pyruvate kinase and through reduced allosteric feed-back inhibition of PEP on phosphofructokinase [14]. On the other hand, a low PEP to pyruvate ratio leads to reduced phosphorylation of glucose transport proteins (EIIA_Crr_ from phosphotransferase system) this way contributing to reduced cAMP formation and to long-term changes in the transcription of CRP-cAMP controlled genes [14, 16] including the down-regulation of genes involved in sugar uptake [17].

### Cells can quickly reduce respiratory energy formation but not glycolytic pathway flux

Altogether, these findings show that cells are prepared to quickly reduce ATP generation e.g. through reduced respiration but need more time to fine-tune catabolic carbon processing thought the glycolytic pathway with their capacities for anabolic processing. For example, the adenylate energy charge returned within minutes after a carbon-upshift imposed on non-recombinant *E. coli* TG1 to the new steady state level while the FBP level needed approx. one hour to decrease again and adapt to the new steady state conditions [11]. In case of permanent perturbations of anabolic carbon processing, e.g. caused by IPTG-induced production of hFGF-2 with recombinant *E. coli* TG1, cells were able to reduce ATP formation to reach again a constant adenylate energy charge within one hour but maintained a permanent high FBP level indicative of an elevated flux through the glycolytic pathway (Figs. 2 and 3).

Metabolites can not only modify fluxes through allosteric modification but also through binding to transcription factors. Thus, metabolic flux control through metabolites can be exerted within seconds through allosteric control of enzyme activity but also long-term through modification of transcription factor activity. For example, the long-term changes in carbon and energy metabolism after IPTG-induced production of hFGF-2 in *E. coli* BL21 (DE3) were mainly mediated through metabolites which e.g. decrease the influence of transcription control through CRP-cAMP and increase the activity of ArcA this way changing the transcriptome and finally reconstructing the cellular protein and enzyme repertoire [17]. These long-term changes in carbon and energy metabolism based on metabolite mediated changes in transcription factor activity are geared towards a reduction of catabolic carbon processing including carbon substrate uptake [17].

Thus, the metabolic response towards induced recombinant protein production can be classified into the following consecutive steps: (i) trying to maintain energetic homeostasis by adjusting (respiratory) energy generation through allosteric control and (ii) trying to reorganize the enzyme repertoire through transcriptional control aiming for reduced catabolic carbon processing. In between glycolysis is “self-accelerating” leading to the excessive accumulation of metabolites such as FBP and pyruvate. Under conditions of compromised anabolic capacities, caused for example by excessive recombinant gene transcription, cells might not always be able to reorganize their metabolic enzyme repertoire as required for reduced carbon processing.

### How to improve recombinant protein production based on the findings above?

The situation described above may have some resemblance to the construction of a house: workers are bringing bricks and cement in a certain rate (catabolism) and other workers are constructing the walls (anabolism). If “something” disturbs or slows down the process of wall construction, the situation gets worse if the other workers continue to bring bricks and cement in the same now too fast rate. Solving the problem is possible through different means, for example, by reducing the speed bringing bricks and cement and/or by reducing the disturbing impact.

Transferring this simplified solution to recombinant protein production can be done, for example, by reducing the carbon feed in controlled fed-batch cultures, as has been shown for *E. coli* BL21 (DE3) [4, 18] as well as for the K12 strain TG1 [19]. Moreover, improved host strains with genetic manipulations affecting carbon metabolism have been constructed although most of them with the initial aim to improve energy generation and/or preventing acetate formation e.g. [20–23]. Interestingly, successful strain constructs with improved protein production properties also exhibited reduced growth rates or glucose uptake rates in the absence of induced protein production suggesting that the beneficial effect is not improved energy generation but the generation of more balanced growing hosts which grow slower and often reach higher biomass concentrations.

The other approach to minimize the metabolic burden is related to the reduction of the “disturbing impact”. Here, most approaches are related to reducing recombinant gene expression, for example by decreasing gene copy numbers, promoter strength or inducer concentration e.g. [24–26]. The exact nature of the “disturbing impact” of enhanced recombinant gene transcription on cell growth is not yet known. However, it is clear that enhanced transcription of genes with different sequences but still encoding the same protein can lead in same cases to severe and in others only to marginal growth inhibition [6, 27, 28]. Moreover, transcription without translation and even the generation of short transcripts can be growth inhibitory [5]. Thus, rational and general approaches to understand and tackle this side of the recombinant protein production associated growth inhibition are still missing and might continue to be unpredictable and different from case to case.

## Conclusions

The cellular response towards recombinant protein production is certainly multifaceted and depends on pre- and post-production conditions as well as on the properties of the recombinant transcript and translated recombinant protein. Recent findings corroborate the hypothesis that the recombinant protein production associated metabolic burden is not the result of limited energy generation but on the opposite an insufficient attempt to reduce catabolic carbon processing because of insufficient carbon withdrawal in anabolic pathways. This response can thus be interpreted as a mal-adaptation of *E. coli* to the non-natural situation of forced recombinant gene expression and/or persistent carbon excess.

## Materials and methods

### Strain, plasmids, medium, culture conditions and basic analytical methods

The *E. coli* K12 strain TG1 (DSM6056) was used as host [29]. The construction of the plasmid pJHLbFGF encoding human basic fibroblast growth factor under control of the *tac*-promoter is described elsewhere [30]. The parental plasmid pJHLΔ does not contain a gene downstream of the *tac*-promoter. Cells were grown on a defined medium containing (per liter) 15 g glucose in bioreactor cultures or 10 g glucose in shake flask (pre)-cultures, 8 g (NH_4_)_2_SO_4_, 0.8 g (NH_4_)_2_HPO_4_, 2.7 g KH_2_PO_4_, 1 g MgSO_4_·7H_2_O, 12 mg Fe(III)citrate, 0.5 mg CoCl_2_·6H_2_O, 3 mg MnCl_2_·4H_2_O, 0.3 mg CuCl_2_·2H_2_O, 0.6 mg H_3_BO_3_, 0.5 mg Na_2_MoO_4_·2H_2_O, 1.6 mg Zn(CH_3_COOH)_2_·2H_2_O, 350 mg citric acid·H_2_O, 1.7 mg EDTA, 4 mg thiamine, and 40 mg ampicillin. Cultivations were carried out in a 2 L bioreactor (Type SGI 7 F-Set2, Setric Genie Industriel, Toulouse/France) at 30 °C, pH 6.6 and a stirring speed of 800 rpm and constant aeration rate of 1.33 vvm. Induction of hFGF-2 synthesis was carried out by the addition of IPTG to a final concentration of 5 mmol L^−1^ when the biomass concentration reached 3 g L^−1^. The control strain TG1:pJHLΔ was grown under identical conditions. Cell growth was monitored by measurement of the absorbance at 600 nm (OD_600_ 1 corresponding to approx. 0.5 g/L dry cell mass). Off-gas data (Oxygor 6 N, Unor 6 N Maihak AG, Hamburg) were used to calculate carbon dioxide and oxygen transfer rates as described previously [31]. For glucose analysis, the YSI 2300 STAT PlusTM glucose & lactate analyzer (YSI Life Sciences, USA) was used. Acetate and pyruvate concentrations were determined using enzymatic kits (Roche Diagnostics). For determination of soluble and insoluble product fractions, cells were disrupted by sonication and the amount of hFGF-2 in each fraction determined by SDS-PAGE analysis.

### Sample preparation and intracellular metabolite analysis

Extraction of intracellular metabolites was carried out as described previously [11, 31] using a fast sampling device and spraying approx. 5 mL cell suspension into a vacuum-sealed test tube containing 1 mL perchloric acid (70%, − 25 °C). After a freeze-thaw cycle between 0 and − 25 °C, the pH of the extract was adjusted on ice to pH 7.2–7.4 using KOH, followed by centrifugation (15 s, 12,000×*g*, centrifuge 5417, Eppendorf, Hamburg, Germany) and filtration (0.45 μm, Millipore, Schwalbach, Germany). For measurement of 3-phosphoglycerate (3PG) the pH of the sample was adjusted to pH 3.5–4.0 with KOH. AMP, ADP, and ATP were analyzed by ion-pair HPLC [32]. All other metabolites were quantified by enzymatic analysis [33]. Since pyruvate was also excreted into the medium, its intracellular concentration was determined by subtracting the extracellular from the total pyruvate concentration. Volumetric concentrations of intracellular metabolites given are related to the cell volume of *E. coli* determined as 2.15 mL g^−1^ [34].

## Data Availability

All data generated or analyzed during this study are included in the published article.
